# Solving the global climate crisis: the greatest health opportunity of our times?

**DOI:** 10.1186/s40985-016-0047-y

**Published:** 2016-12-07

**Authors:** Jonathan A. Patz

**Affiliations:** Global Health Institute, University of Wisconsin, Madison, WI USA

**Keywords:** Public health, Climate change, Carbon policy, Active transport, Diet, Co-benefits

## Abstract

Today’s substantial global health gains are being undermined by the threat of climate change. Ironically, the actions required to confront the climate crisis represent possibly the largest public health opportunity in more than a century. Health benefits from improved air quality may far outweigh the cost of clean energy investments. Upward trends in chronic diseases are now occurring throughout the world. Herein lies even more golden opportunities for public health through the following: first, adopting more alternative modes of transportation, especially those that promote “active transport” by foot or by bicycle, in combination with effective public transportation; and second, by reducing meat in the diet. In essence, there is no better time to focus on health as central in the climate negotiations; and in so doing, may we move faster and further with effective actions on climate change and the subsequent health benefits that will arise from a low-carbon society.

## Main Text

According to reports of the Intergovernmental Panel on Climate Change (IPCC), the Lancet Commission on Health and Climate Change and many more, today’s substantial global health gains are being undermined by climate change [1]. A wealth of evidence shows that global health and global climate and ecological conditions are inseparable. Healthy human populations simply cannot be sustained on a sick planet. The global climate crisis therefore demands a rapid change in policies and collective actions to divert our current path toward a 7 °C warmer world by the end of this century. Unfortunately, environmental and economic arguments, while important, are not moving climate change policies quickly enough. Caring about our own health tends to supersede all other priorities. Therefore, focusing on the problems of and solutions to climate change through a health lens compliments not only the environmental and economic efforts but also, most importantly, a health framing that can bring more focus and resolve to the global climate crisis.

It might seem like a paradox, but the actions taken to confront climate change today represents possibly the largest public health opportunity in more than a century. Consider the following realities: (1) The World Health Organization (WHO) estimates seven million deaths are attributed to air pollution every year, (2) rates of obesity and chronic diseases are rising in nearly all regions of the world, and (3) greenhouse gas emissions—responsible for the global climate crisis—rose the fastest (~2%/year) in the past decade, approximately twice the rate from the period between 1970 and 2000 according to the IPCC.

Fossil fuel combustion is the common link to these three realities. Fossil fuel combustion accounted for 78% of the total increase in carbon dioxide between 1970 and 2010 (with deforestation comprising the balance of emissions). Of course, burning oil, gas, and coal also release pollutants such as fine particulates, e.g. PM_2.5_, known to be harmful to human health. Cleaner energy can help reduce the heating of the planet, while saving lives from air pollution. Greenhouse gas mitigation strategies could avoid an estimated one to four million deaths annually by 2050 [2]. Health benefits may far outweigh the cost of clean energy investments. For example, in the United States of America, monetized health benefits associated with improved air quality can offset between 26 to 1050% of the cost of US low-carbon policies [3]; in other words, the value of health dividends could swamp the costs of striving for an energy-efficient, low-carbon economy. This should be of no surprise given the US Environmental Protection Agency (EPA) estimates of a $30 return for every dollar invested in reducing air pollution through the Clean Air Act. And health benefits will be even larger in highly polluted cities across other regions of the world.

Upward trends in obesity and chronic diseases, such as diabetes and heart disease, are now occurring throughout the world [4], as Western lifestyles with automobile-dependent transportation and meat-based diets are being pursued. Herein lies even more opportunities for public health through the following: first, adopting more alternative modes of transportation, especially those that promote “active transport” by foot or by bicycle, alongside of effective public transportation; and second, by reducing meat in the diet.

Studies from across the world show marked health benefits from active transport [5]. Active transport in Shanghai, China could reduce colon cancer risk by over 44%.

Bike commuting in London could lower ischemic heart disease by 10 to 19%.

In the USA, comparing cities with the highest versus the lowest levels of active transport, obesity rates are 20% lower and diabetes rates are 23% less, and switching short car trips to bike trips would save 1300 lives annually for just one region of the USA. Bicycling commuters in Copenhagen have a 39% reduction in mortality rate compared to non cycling commuters. While less studied, rapidly growing newer cities, especially in Africa, provide especially unique health opportunities from urban planning; in these locations, we have an opportunity to design cities for the health of people, rather than simply for the flow of motorized traffic.

Diet and food systems represent another key focus area for dual benefits to our health and the environment. In the United Kingdom, if 50% of meat and dairy in the diet were replaced by fruit, vegetables, and cereals, greenhouse gas emissions might drop by 19%, while at the same time potentially 30,192, to 43,592 deaths could be averted per year by the reduction of saturated fat in the diet [6]. However, meat in the developing world provides essential protein and micronutrients—so this recommendation is geared primarily to “supersized” industrialized countries.

## Conclusions

Current rates of chronic disease alongside continued rising trends in fossil fuel-based energy consumption that are causing today’s global climate crisis present daunting risks to civilization. The interdependence of these challenges, however, affords an enormous opportunity to solve both simultaneously. Following the landmark 21st Conference of the Parties (COP21), with the Paris Agreement now officially in force, attention is on COP22 to accelerate actions to mitigate greenhouse gas emissions. There is no better time to focus on health as central in the negotiations; and in so doing, may we move faster and further with effective actions on climate change and the subsequent health benefits that will arise from a low-carbon society.

## Abbreviations

COP: Conference of the Parties
EPA: Environmental Protection Agency
IPCC: Intergovernmental Panel on Climate Change
PM: Particulate matter
US: United States of America
WHO: World Health Organization
